# Helicopter Journals

**DOI:** 10.1128/mBio.01046-16

**Published:** 2016-07-26

**Authors:** Antonio Cassone

**Affiliations:** Polo d’Innovazione della Genomica, Genetica e Biologia, University of Perugia, Perugia, Italy

## LETTER

The impact factor mania, also called impactitis, continues to play an improper role in the choices made by most investigators as to which journals to submit their research articles in the biomedical fields, and still worse, in the decisions taken by governing bodies and research grant allocators, as highlighted in a recent *mBio* editorial (1). Other factors, however, are threatening the scientific community and, on a broader scale, that part of the enterprise which continues to credit published science as the source of correct information for the basis of important decisions impacting human life. Helicopter journals are one such factor. But let me start from the viewpoint of economic issues.

Heavy economic stresses are known today to affect most parts of the developed world, in particular some European countries. Low growth and investment rates, then recession and high risks of deflation are giving strength to an old proposal launched by the Nobel Prize winner Milton Friedman, the famous monetary economist and founder of the Chicago School of Economics. He suggested giving people free money to spend as an alternative to the quantitative easing adopted by national banks, most recently by the Central European Bank. For this proposal, he coined the term “helicopter money,” reminding us of the famous Bible episode about the fall of manna from heaven (2). Money from the heavens is currently debated and also believed by some renowned economists to be a useful intervention to fight deflation. This is challenged by the majority of other economists and, possibly, will remain only an academic theory (3).

What is not a theory is the explosive growth of new journals offering to publish technical and scientific papers. They too, and copiously, fall from the celestial cyberspace to our desktop. Consequently, I am taking the liberty of calling them “helicopter journals” although, as will be seen, what falls to our desktops is really not a form of manna.

There is no day, I would say no moment, that any active researcher is not overwhelmed by e-mails advertising new, in most cases online, journal publishers repeatedly exhorting the researcher to submit papers. In many cases, they offer a discounted rate for the cost of publication, even of the “submit two papers at the cost of one” kind, in the best spirit of ShopRite or Walmart (without offense to those esteemed supermarket brands)! Some of these new journals have unbelievable names, such as *Journal of Bacteria*, *Virus and Fungi* (what about protozoa?) or the *Journal of Infectious and Noninfectious Diseases* (thank god, health is out!). This is coupled with an equally pressing number of “honorable speaker invitations” to hundreds of meetings around the world, usually without any relation to the field of interest of the message recipient, without any offer to cover the expenses of the trip and accommodations, but of course with an offer of free speech. Just today, while writing this letter, I have received, among others, an invitation to speak at a meeting on anatomy and another to speak at a meeting for chemical engineering!

At this explosive daily rate, the numbers of field journals (already several thousands in the biomedical areas) will soon, and ridiculously, outnumber those scientists actively working in that discipline. Usually, most of these new journals guarantee, sometimes in a rather explicit way, article publication without a real process of peer review. In other cases, the absence of a true peer review process is implicit, for instance, when a journal promises the authors a decision in 7 days, a time that makes impossible any accurate review of the paper, even with the highest editorial dedication.

Why is this happening? In very few cases, this expansion represents a real scientific need of rearranging or reconsidering the field of interest to better match the present evolution of the field or even to cover a new area of research. This usually happens when changes are fostered by journal-publishing scientific societies. Some journals die, others undergo name changes; this is normal life. When this happens, the value and rigor of the new journals equal or surpass those of the old ones. Overall, calls to submit papers for possible publication is not bad in itself, particularly when they come from old and highly reputed journals that use a fierce peer review system. For instance, the *Journal of Experimental Medicine* has recently encouraged submissions by offering format-neutral initial submissions, a chance for review of the paper independently of its style and format, via a link to BioRxiv (http://jem.rupress.org). That these authors are offered an expanded market of serious publishing space is welcomed.

In most other cases, however, the new offers have a purely commercial reason. With the present digital era, it has become quite inexpensive to produce an online journal: a good server and (perhaps) an underpaid secretary may be enough. Of course, you cannot expect a rigorous, respectful, and accurate reviewing system if the earning appetite is the only motivation. The more papers submitted, the more that are published, and the more the publisher earns. In other cases, new journals are being created by some investigators with or without the support of their societies. The underlying motivation is a desire of increased notoriety within the scientific community or a disguised pride to gain some editorial power, perhaps motivated by the frustrations coming from the lack of visibility or rejection of their articles, a fact that is hardly digested after a certain age and a long career. This is comprehensible but unjustified. Finally, it has also become a question of geographic if not political interest. The very large majority of the most authoritative scientific journals are run by publishers and scientific societies largely located in Western countries (the United States and Europe mostly) and using English as the publication language. While this is almost universally accepted, some are inclined to think that publishers from other continents should come into play, offering low-cost publishing space and better representing the research activity of their nations. This can be fair; nonetheless, worldwide expansion of inexperienced publishers equals journal expansion and increases the risk of unreliable data publications and predation (https://scholarlyoa.com/2016/01/05/bealls-list-of-predatory-publishers-2016/).

Altogether, the pressures created by the offer of publication will inevitably attract more publications and result in a poor quality of what is being published, thus widening the plethora of an already vast and unreliable scientific literature (4). In particular, young investigators under pressure to publish to initiate or accelerate their academic careers are linked to this in support of their request of grants. They might find it convenient to avoid submission to authoritative journals with their usual rigorous but time-consuming peer review and high rate of rejection. In terms of the impact of their studies, some investigators could choose a minor gain with a predictable outcome rather than a greater gain but with an unpredictable outcome. Nonetheless, the rite of passage involved in working through the criticisms of experienced reviewers and editors is an important, in some cases necessary, step for a young researcher, because it promotes self-critical data appraisal before submission for publication and improves the overall quality of their work.

Another perhaps more relevant consequence is the danger that an uncontrollable flow of publications can affect scientific research by decreasing its social credit in an era of easy, direct, or indirect access to what is published and the derived media reports. We are all aware that the quantity of unreliable reports is high and that even the most accurate review system cannot entirely avoid false reports or fraud, as evident from the paper that associated measles vaccination with autism (5). Uncontrolled eagerness to publish incited by the plethora of offers could lower the quality of what is published and bring society to perceive that science journals are something like social media, where anyone can say anything. This equation would seriously detract from the public credibility science still has.

As an example, consider the area of vaccines and vaccinations. Some coarse errors that continue and are being repeated by social media, such as the view that some vaccines are associated with autism or asthma, are rightly and effectively counteracted by rigorous scientific evidence of studies demonstrating the absence of any causal relationship between vaccination and those conditions. My experience is that accurate narration of the outcome of those published studies (6) is usually more persuasive than just highlighting past vaccine successes. Most people still believe that scientific publications inform them with reasonable certainty. It is our duty to ensure that scientific findings continue to be conveyed by journals which apply rigorous publishing criteria. Helicopter journals do not help.
